# Extensive cardiovascular involvement in a young boy with Gaucher’s disease: a case report

**DOI:** 10.1093/ehjcr/ytad456

**Published:** 2023-09-11

**Authors:** Mohammadreza Naderian, Hamid Khederlou, Ali Hosseinsabet, Mojtaba Salarifar

**Affiliations:** Tehran Heart Center, Tehran University of Medical Sciences, North Kargar Street, Tehran, Iran; Tehran Heart Center, Tehran University of Medical Sciences, North Kargar Street, Tehran, Iran; Tehran Heart Center, Tehran University of Medical Sciences, North Kargar Street, Tehran, Iran; Tehran Heart Center, Tehran University of Medical Sciences, North Kargar Street, Tehran, Iran

**Keywords:** Gaucher’s disease, Cardiovascular involvement, Valvular calcification, Supra-valvular aortic stenosis, Case report

## Abstract

**Background:**

Lysosomal storage diseases (LSDs) are rare, progressive, multi-organ disorders caused by inherited enzyme deficiencies. Gaucher’s disease (GD) is the most prevalent form of LSDs.

**Case summary:**

A 19-year-old Caucasian male presented with exertional dyspnoea. Physical examination revealed a Grade III/VI systolic diamond murmur at the heart base and a Grade IV/VI systolic murmur at the apex. Electrocardiogram showed signs of left ventricular hypertrophy (LVH). Trans-thoracic echocardiography (TTE) and trans-oesophageal echocardiography (TEE) demonstrated moderate LVH, severe aortic valve stenosis, severe supra-valvular aortic stenosis, and moderate mitral stenosis with severe degenerative mitral valve regurgitation. Bone marrow biopsy and aspiration confirmed the presence of characteristic Gaucher's cells. The patient underwent the Bentall procedure and mitral valve replacement and was discharged in good condition.

**Discussion:**

Gaucher’s disease exhibits three clinical phenotypes, and cardiovascular involvement is commonly seen in GD Type III. Valvular calcification and ascending aorta involvement are frequent cardiovascular manifestations. Although severe valvular heart involvement is rare in GD, cardiac valve surgery has shown favourable outcomes in previous studies and our case.

**Learning points**

- Gaucher’s disease (GD) can have significant cardiac involvement.
- Calcific degenerative valve disease can lead to stenosis and regurgitation of the heart valves in GD.
- Aortic hypoplasia is a known manifestation of GD.
- Surgical intervention can yield positive outcomes in cases of severe valve involvement in GD patients.

## Introduction

Lysosomal storage diseases (LSDs) encompass a group of rare, progressive, multi-organ, and heterogeneous abnormalities caused by inherited enzyme deficiencies. These disorders comprise over 50 genetic diseases.^1^ Among them, Gaucher’s disease (GD) stands as the most prevalent form of LSDs.^2^ Gaucher’s disease results from mutations in the glucocerebrosidase (GBA) gene, leading to a deficiency of acid beta-glucosidase (glucocerebrosidase). The subsequent accumulation of glucosylceramide within macrophage lysosomes adversely affects cells of the reticuloendothelial system, ultimately impacting the liver, spleen, and bone marrow. Furthermore, the involvement of central nervous system and cardiovascular system has been documented.^3^ Studies have identified various cardiac manifestations in GD patients, including cardiomyopathy, chronic heart failure, sudden cardiac death,^4^ constrictive pericarditis,^5^ and valvular heart conditions such as aortic valve (AV) calcification,^6^ mitral valve (MV) insufficiency, and tricuspid valve (TV) insufficiency.^7^ In this case report, we present a young boy with extensive cardiovascular involvement, which posed challenges in both the diagnostic process and management. The notable presence of extensive cardiac valve calcification, coupled with multi-system involvement and an atypical presentation, motivated us to document this patient’s case.

## Summary figure

**Table:** 

Timeline	Events
**Initial presentation**	Dyspnoea on exertion since 2 months prior. Grade III/VI systolic diamond murmur on the heart base and a Grade IV/VI systolic murmur in the apex of the heart.
**Initial para-clinic investigations**	
Day 1	Electrocardiogram revealed left ventricular hypertrophy.
Day 1	Trans-thoracic echocardiography and trans-oesophageal echocardiography showed moderate left ventricular hypertrophy, severe aortic valve stenosis, severe supra-valvular aortic stenosis and moderate mitral stenosis with severe degenerative mitral valve regurgitation.
Day 2	Laboratory investigation showed thrombocytopaenia with a platelet count of 62 000 × 10^3^/µL.
Day 7	The bone marrow biopsy and aspiration showed characteristic findings of Gaucher’s cell.
**Day 9**	The patient underwent Bentall procedure and mitral valve replacement.
**Day 18**	He was discharged.
**Day 30 after cardiac surgery**	The patient hospitalized with COVID-19 infection.
**Day 36 after cardiac surgery**	The patient was recovered and discharged.
**Follow-up visits 1, 3, and 6 months after cardiac surgery**	Both AV and MV prosthetic valves are as good leaflet motion and acceptable gradients in TTE.

## Case presentation

A 19-year-old Iranian male of Caucasian ethnicity sought treatment at a tertiary centre specializing in cardiovascular diseases. The patient’s chief complaint was dyspnoea on exertion, which he had been experiencing for the past 2 months. His condition corresponded to New York Heart Association functional Class II (NYHA Class II).

The patient denied any additional symptoms. His medical history revealed a diagnosis of hypothyroidism, and he reported no known genetic or congenital disorders in his parents. During the initial physical examination, the patient exhibited short stature (height: 135 cm and weight: 53 kg), but his blood pressure, heart rate, oxygen saturation, and body temperature were within the normal range.

Upon auscultation, a characteristic murmur consistent with aortic stenosis was detected. The murmur was described as high-pitched, crescendo–decrescendo in nature, and exhibited a mid-systolic ejection pattern. It was graded as III/VI and best heard at the heart base. Additionally, a Grade IV/VI holosystolic (pansystolic) murmur was noted, audible at the apex with the diaphragm of the stethoscope, particularly when the patient assumed the left lateral decubitus position. This finding was suggestive of mitral regurgitation (MR). Symmetrical pulses were observed in both the upper and lower limbs, and a palpable spleen was detected ∼4 cm below the costal margin. No other significant abnormalities were identified during the examination. The patient was on a regular daily dosage of levothyroxine (0.1 mg).

**Figure 1:**
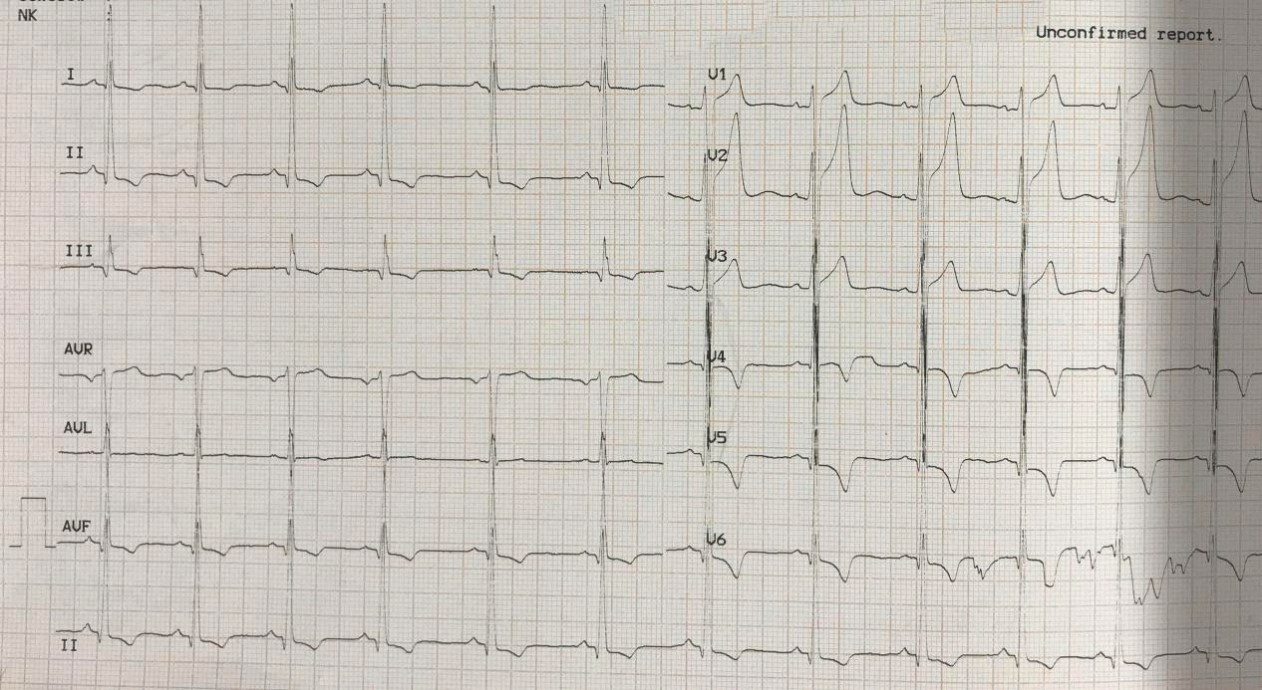
The electrocardiogram at presentation revealed normal sinus rhythm with a normal axis, accompanied by high-voltage QRS complexes and secondary ST segment and T-wave changes in Leads I, II, aVL, and V3 to V6, indicative of left ventricular hypertrophy.

At the time of presentation, the electrocardiogram (ECG) exhibited changes indicative of left ventricular hypertrophy (LVH) (*Figure 1*).

**Figure 2:**
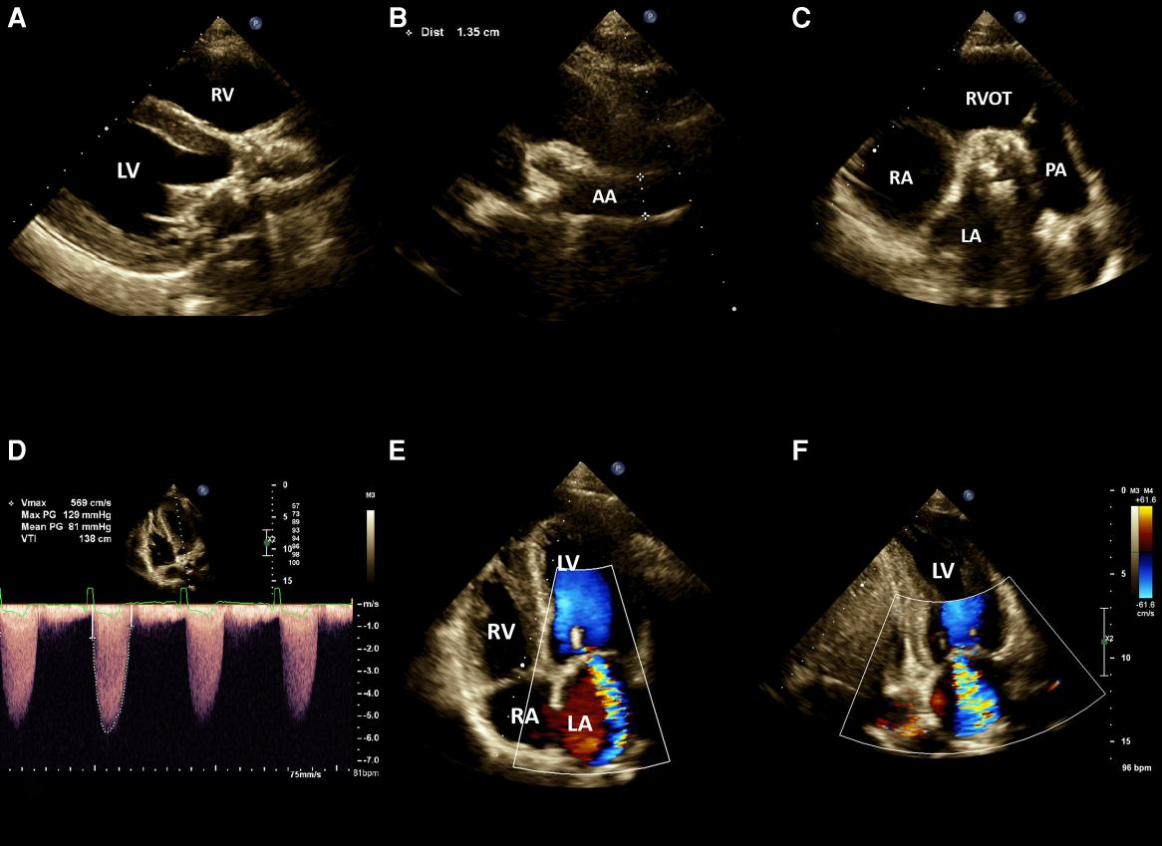
Trans-thoracic echocardiography demonstrated a normal left ventricular size with a moderate symmetrical increase in left ventricular wall thickness, suggestive of concentric left ventricular hypertrophy, as observed in the parasternal long-axis view (*A*). The examination also revealed a small aortic root and ascending aorta (∼14 mm) and a small aortic valve orifice with significant calcification and a marked increase in trans-valvular velocity (5.7 m/s), indicative of both valvular and supra-valvular aortic stenosis (*B*–*D*). In the apical two- and four-chamber views, there was severe trans-valvular mitral regurgitation (*E* and *F*). AA, ascending aorta; LA, left atrium; LV, left ventricle; PA, pulmonary artery; RA, right atrium; RV, right ventricle; RVOT, right ventricular outflow tract.

**Figure 3:**
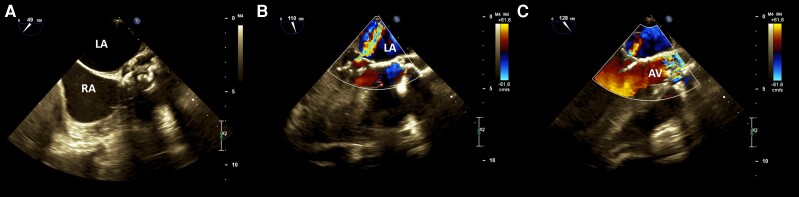
Trans-oesophageal echocardiography in short and long-axis views showed calcified aortic valve’s cusps with the extension of non-coronary cusp’s calcification to the anterior mitral valve leaflet, resulting in some degree of mitral stenosis (*A* and *B*). The same as the trans-thoracic echocardiography, again we can prove the severe regurgitation of blood through the mitral valve and significant increase in velocity of blood while passes through the aortic valve (*C* and *D*). AV, aortic valve; LA, left atrium; RA, right atrium.

The patient underwent trans-thoracic echocardiography (TTE) initially, and subsequently, trans-oesophageal echocardiography (TEE) was performed due to abnormal findings observed in the TTE (*Figures 2* and *3*). Trans-thoracic echocardiography and the complementary TEE examinations revealed a LV ejection fraction of ∼65% and moderate LVH. Furthermore, the AV was observed to be thickened with severe calcification of the AV cusps, resulting in severe AV stenosis. The maximum velocity (*V*_max_) across the AV was measured at 5.6 m/s, and the AV area was determined to be 0.84 cm^2^.

The severe calcification observed in the proximal region of the under-developed ascending aorta and aortic arch led to the presence of severe supra-valvular aortic stenosis. Additionally, the calcification extended to the aortomitral continuity and anterior MV leaflet, resulting in moderate mitral stenosis and severe degenerative MR. The echocardiographic findings indicated a mean gradient across the MV of 10 mmHg, a vena contracta of 7.4 mm, a regurgitant volume of 84 mL/beat, and an effective regurgitant orifice area (EROA) of 0.43 cm^2^. It is noteworthy that the patient’s heart rate during the echocardiographic examination for the assessment of the MV gradient was recorded as 82 b.p.m. The initial laboratory investigations revealed normal results, except for thrombocytopaenia, with a platelet count of 62 000 × 10^3^/µL (150 000–450 000 × 10^3^/µL). Thyroid function tests were within normal limits. Microscopic examination of the peripheral blood smear showed thrombocytopaenia, along with a few foci of platelet aggregation. The red blood cell count and white blood cell count appeared normal, both in terms of count and morphology.

Abdominal ultrasound revealed an abnormal finding of splenomegaly, with a span measuring 180 mm. Further imaging with multi-detector computed tomography (CT) of the aorta, cervical, and cerebral arteries confirmed the previously visualized findings from the echocardiography examination (see Supplementary material online, *Movie S1*). It was observed that the aortic arch and descending aorta also exhibited hypoplasia, similar to the under-developed ascending aorta with an average diameter of 15 mm. The assessment of the patient’s cardiovascular system included the examination of the aortic arch branches, abdominal aorta, and its branches, as well as the cervical and cerebral arteries. These blood vessels were found to be patent and exhibited a normal anatomical structure. Additionally, the coronary CT angiography (CTA) scan indicated a normal course and no significant luminal stenosis in the coronary arteries. However, it was noted that there was calcification present in the left circumflex artery.

**Figure 4:**
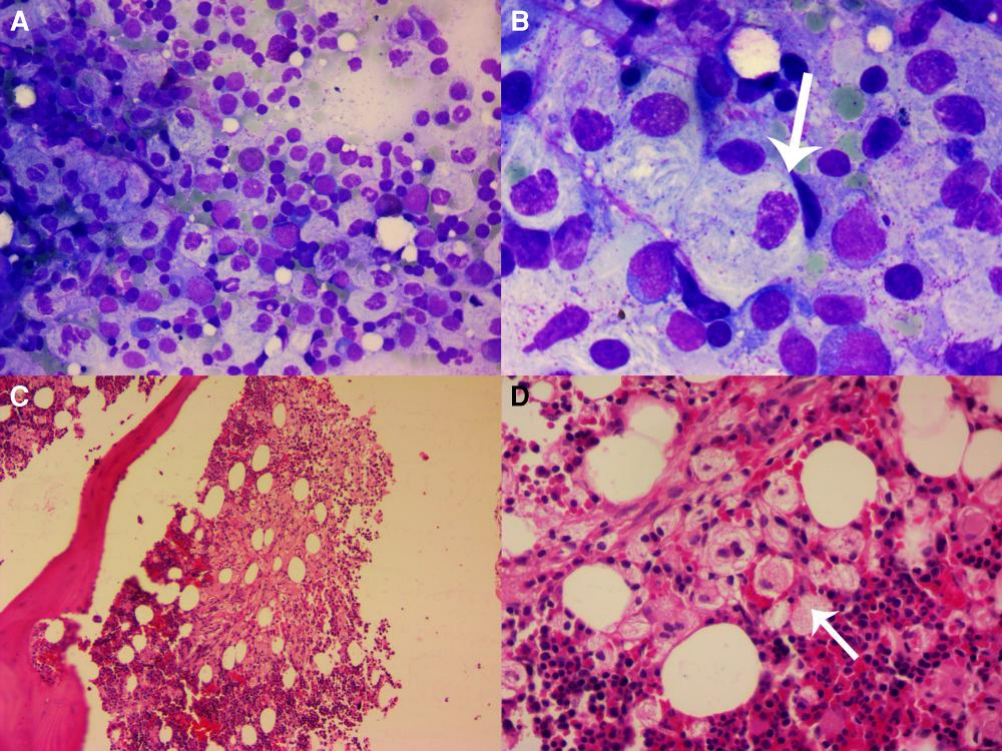
Bone marrow aspiration (*A* and *B*) and biopsy (*C* and *D*) reveal the presence of numerous atypical histiocytes arranged in sheets and clusters. These histiocytes (indicated by white arrows) exhibit abundant fibrillary or striated cytoplasm and possess nuclei with a bland appearance, which are characteristic features of Gaucher cells. The histological slides were stained with haematoxylin and eosin, and the magnification levels are as follows: *A*: ×400; *B*: ×1000; *C*: ×100; and *D*: ×400.

Considering the extensive cardiovascular involvement in the patient and the scarcity of similar cases documented in the literature, a heart team was convened. After careful deliberation, it was decided that surgical intervention was necessary due to the worsening dyspnoea experienced by the patient. Prior to the open-heart surgery, a bone marrow aspiration and biopsy were performed. The results of the bone marrow biopsy and aspiration revealed the presence of clusters of eosinophilic histiocytes exhibiting a fibrillar or striated appearance and characterized by thin nuclei. These findings were consistent with Gaucher cells, indicative of GD (*Figure 4*).

The patient underwent surgery, which the Bentall procedure was performed by the surgeon using a mechanical composite St-Jude #21 valve. Additionally, MV replacement was carried out using a mechanical St-Jude #27 valve. Septal myectomy was also performed, due to the high LV outflow tract pressure gradient (LVOT PG) and high LV end-diastolic pressure. Following the operation, a post-operative echocardiography examination revealed preserved LV systolic function, as well as well-functioning prosthetic valves in both the AV and MV. The echocardiogram also indicated good leaflet motion and acceptable gradients across the prosthetic valves.

Upon discharge, the patient has prescribed warfarin, aspirin 80 mg daily, and bisoprolol 1.25 mg daily as part of their post-operative management. However, 1 month after the surgery, the patient contracted a COVID-19 infection. Fortunately, they recovered after receiving a course of intravenous glucocorticoid therapy. During the follow-up visits at 1, 3, and 6 months, the patient’s symptoms improved, and they were in good overall condition. It is important to note that the patient underwent enzyme replacement therapy with imiglucerase (Cerezyme) at a monthly dosage of 1200 units. As a result of this treatment, the patient’s platelet count remained within the range of 100 000–130 000 × 10^3^/µL. Therefore, splenectomy was not deemed necessary in this case.

## Discussion

Lysosomal storage diseases encompass a broad spectrum of over 50 genetic diseases, several of which exhibit cardiac involvement, including GD, Fabry disease, and mucopolysaccharidoses.^1,8^ Gaucher’s disease is classified into three clinical phenotypes. Gaucher’s disease Type 1 (adult) constitutes the majority, accounting for over 90% of GD cases, and is commonly observed in the Ashkenazi Jewish population. Patients with Type 1 GD typically experience non-neural manifestations. Type 2 GD (infantile) represents the most severe neuropathic form, leading to premature death during childhood. Type 3 GD (childhood) is an intermediate form between Types 1 and 2, affecting both the nervous and reticuloendothelial systems.^9^ While the gold standard for GD diagnosis is the measurement of glucocerebrosidase enzyme activity, it is often confirmed through the identification of Gaucher’s cells in bone marrow aspirate/biopsy (BMA/B) samples.^10^ In the case of our patient, a BMA/B was also performed, revealing the presence of characteristic Gaucher’s cells, supporting the diagnosis of GD. Considering the patient’s involvement of the reticuloendothelial system and the age of disease onset, GD Type 3 is considered in this study.

Leopard syndrome was initially considered as a possible diagnosis for this patient due to the presence of multiple stenoses in the left cardiovascular system. However, this diagnosis was ultimately ruled out based on pathology findings and the specific pattern of valvular involvement observed. The patient’s clinical presentation and examination findings were not consistent with the characteristic features of Leopard syndrome, leading to the exclusion of this condition as a potential diagnosis. Also, hypertrophic cardiomyopathy was one of the differential diagnoses, which was ruled out according to investigations and pathophysiological diagnosis.

Cardiovascular involvement is indeed observed in GD Type III, as documented in previous studies.^9^ The age of onset for this type of GD can vary, ranging from childhood to the seventh decade of life, with ∼70% of cases manifesting before the age of 20, which aligns with the presentation of our patient in this study.^11^ Although cardiovascular involvement in GD is rare, it can be potentially fatal. Valvular and vascular calcifications, as well as involvement of the ascending aorta, are more commonly reported in cases of GD,^5,6^ which is consistent with the findings in our patient. However, the exact pathophysiology underlying the development of valvular, vascular, and myocardial involvement in GD remains unknown.^11^ Further research is needed to elucidate the mechanisms involved in these cardiovascular manifestations.

In addition, we observed extensive calcification of the left circumflex artery in the coronary CTA of our case. Similarly, previous studies have reported MV involvement, specifically MV, and stenosis, which is consistent with our findings.^7,8,11^ Interestingly, our study revealed aortic involvement characterized by both valvular and supra-valvular stenosis without significant regurgitation, a pattern not previously documented in GD patients.^8,11^ In a limited number of GD cases, severe involvement of cardiac valves has been observed. Similar to our patient, these individuals underwent mitral and AV replacement surgery and experienced favourable outcomes during follow-up.^12^ Cardiac valve surgery in GD has been demonstrated to be a successful procedure with positive outcomes, as supported by previous studies and our case.^12^ While LVH has been reported in other LSDs,^1^ it has not been commonly observed in GD cases according to previous studies, unlike in our patient. Additionally, pulmonary hypertension has been documented in some patients with GD Type III,^13^ which aligns with the presence of this condition in our patient. Enzyme replacement therapy has shown efficacy in reducing the severity of the disease and alleviating certain manifestations, including thrombocytopaenia, as evident in our patient.^14^

## Conclusion

Gaucher’s disease can exert a significant impact on the heart, manifesting as various forms of involvement, including valvular, muscular, and vascular abnormalities.

## Supplementary Material

ytad456_Supplementary_Data

## Data Availability

The data underlying this article will be shared on reasonable request to the corresponding author.

## Lead author biography

Mohammadreza Naderian graduated from medical school in 2017 and began his residency in cardiology at the Tehran Heart Center (THC), a renowned tertiary centre in Iran, specializing in treating cardiovascular diseases. Completing his training in 2021, he commenced his career as an assistant professor of cardiology at the hospital mentioned above, affiliated with the Tehran University of Medical Sciences (TUMS). Currently, he holds the position of a post-doctoral research fellow in cardiovascular genomics medicine at Mayo Clinic, located in Rochester, USA.

## Supplementary material

**Consent:** The authors affirm that they have obtained written consent from the patient in accordance with COPE (Committee on Publication Ethics) guidelines, allowing for the submission and publication of this case report, including accompanying images and associated text.

**Funding:** None declared.
